# Pain of Temporomandibular Disorders: From Etiology to Management

**DOI:** 10.1155/2018/4517042

**Published:** 2018-06-20

**Authors:** Mieszko Wieckiewicz, Yuh-Yuan Shiau, Klaus Boening

**Affiliations:** ^1^Department of Experimental Dentistry, Faculty of Dentistry, Wroclaw Medical University, 50425 Wroclaw, Poland; ^2^Department of Prosthetic Dentistry, School of Dentistry, National Taiwan University, Taipei City 100, Taiwan; ^3^Department of Prosthetic Dentistry, Faculty of Medicine, Dresden University of Technology, 01307 Dresden, Germany

Temporomandibular disorders (TMD) are musculoskeletal disorders with primary symptoms of pain localized to the face and temple and limitation of jaw function [1]. TMD is the second most common musculoskeletal disorder after chronic low-back pain causing pain and disability of the body [2]. TMD is not a disease entity but rather a broad term comprising many disease entities. In recent years, there has been a very intensive and varied development of research on etiology, epidemiology, symptomatology, and management of TMD-related pain. According to the widely used definition introduced by the International Association for the Study of Pain, pain is “an unpleasant sensory and emotional experience associated with actual or potential tissue damage, or described in terms of such damage” [3].

The symptoms of TMD can include pain or tenderness of temporomandibular joints (TMJs) area, clicking, popping, or crepitus in the TMJs, limited jaw movements, masticatory muscle pain, headache, tinnitus, impaired hearing, and earache. It had been proved that TMD are related to multiple causes and biopsychosocial model which is a broad view that attributes disease outcome to the intricate, variable interaction of biological factors, psychological factors, and social factors [4].

The understanding and management of TMD pain usually require a multidisciplinary approach and that is the main target of this special issue. Interdisciplinary therapeutic possibilities should concern TMJs and neuromuscular structures of the masticatory system and supportive tissues at head and neck areas. Moreover, the involvement of psychological and social factors makes physical approaches insufficient. Based on this understanding, the editors of this special issue have invited researchers with wide scope of knowledge and study on pain of TMD.

Original and review articles related to the pain of TMD associated with basic and clinical studies on multiple branches of medicine and novelty in management have been welcome. The published papers introduce the multidisciplinary problem to the reader. The aim of this issue was also to show the novelties in management of TMD pain. A group of articles describe the etiology of the disorders, as well as its epidemiology, accurate diagnostics, and management methods. The special issue aims at collecting a multidisciplinary approach on the pain of temporomandibular disorders in basic and clinical aspects. Current views included in this issue will hopefully allow us to assess a perspective of the TMD-related pain and to develop and familiarize with new research related to the most recent multidisciplinary view points.

*Mieszko Wieckiewicz* *Yuh-Yuan Shiau* *Klaus Boening*
